# A Swiss nationwide survey shows that dual guidance is the preferred approach for peripheral nerve blocks

**DOI:** 10.1038/s41598-019-45700-3

**Published:** 2019-06-24

**Authors:** Markus M. Luedi, Vanessa Upadek, Andreas P. Vogt, Thorsten Steinfeldt, Urs Eichenberger, Axel R. Sauter

**Affiliations:** 1 Department of Anaesthesiology and Pain Medicine, Inselspital, Bern University Hospital, University of Bern, Bern, Switzerland; 2 Department of Anaesthesiology, Diakonie-Klinikum Schwäbisch Hall, Schwäbisch Hall, Germany; 3 Department of Anaesthesiology, Intensive Care and Pain Therapy, Balgrist University Hospital, University of Zurich, Zurich, Switzerland; 4 Division of Emergencies and Critical Care, Department of Anaesthesiology and Department of Research and Development, Oslo University Hospital, Oslo, Norway

**Keywords:** Medical research, Health care

## Abstract

Ultrasound has significantly increased safety and effectiveness in regional anesthesia. However, little is known about its clinical use. We studied clinical approaches currently used by anesthesiologists, conducted a nationwide survey, and analyzed data collected in ordered logistic regression models. All active members of the Swiss Society for Anaesthesiology and Resuscitation (SSAR/SGAR) were asked to participate. Reported practice in nerve localization, safety, and techniques used for peripheral nerve blocks (PNB) were main outcome measures. Experience ranged from 3 to >30 years. The mean number of block techniques mastered was 11.5 ± 5.9. Standard monitoring was regularly used, whereas sterile coats were less frequently used by anesthesiologists who self-estimated a higher level of expertise in PNB (ordered logit coefficient −0.05, 95% CI −0.07 to −0.02, *P* < *0.001*; pseudo r2 = 0.019; probability > Chi2 = 0.02). The more self-estimated expertise anesthesiologists had, the less likely they were to use nerve stimulation in combination with ultrasound (dual guidance) (ordered logit coefficient −0.31; 95% CI −0.85 to −0.03: *P* = 0.03; pseudo r2 = 0.007; probability > Chi2 = 0.05). The high share of reported standard monitoring meets the recommendations of the Helsinki Patient Safety Declaration. Dual guidance appears to be the preferred approach for safely localizing nerves for PNB in Switzerland.

## Introduction

Suitable equipment and technique are keys in providing safe and effective regional anesthesia. While the implementation of ultrasound has significantly increased both safety and effectiveness, successful administration of regional anesthesia depends on additional factors such as physicians’ training, patient and block selection^1^. In order to avoid regional anesthesia-related peripheral nerve injury, reliable nerve localization is of paramount importance^2^.

However, despite sophisticated simulation opportunities, numerous publications, and extensive discussion, little is known about anesthetists’ current clinical practice and the technical approaches they use to localize peripheral nerves for regional anesthesia.

Therefore, we aimed to study the clinical practices and technical approaches currently used by anesthesiologists to provide regional anesthesia. To obtain robust data, we opted for a nationwide survey in Switzerland, a culturally diverse country with four national languages.

We hypothesized that a nationwide survey would shed light on current practice in nerve localization, safety, and technical approaches used for PNB. Furthermore, we considered whether the choice of equipment and procedures (such as nerve stimulation and ultrasound, sterile preparations, periprocedural analgesia, sedation, and clinical approaches) depends on factors such as the anesthetist’s experience and expertise, the hospital setting, and the country’s language region.

## Results

Between June 8 and July 6, 2018, 422 (37.7%) anesthesiologists (262 male, 157 female, 3 unspecified) participated in the survey. Ninety-eight (23%) of the participants were working at university hospitals, 100 (24%) at cantonal hospitals (largest non-university hospitals in Switzerland run by the cantonal governments, some of them level 1 centers), 81 (19%) at regional hospitals, nine (2%) at district hospitals, and 104 (25%) at private hospitals. Twenty-six (6%) of the anesthesiologists were working in other institutions and four (1%) participants did not give details of their affiliations.

### Demography

Switzerland is known for its cultural differences between German-speaking, French-speaking, Italian-speaking, and Romansh-speaking people, not only in social life but also in medical practice. Thus, we considered the region and language of the participants to be potentially relevant information. The main language spoken in the hospital was German (322 participants, 77%), French (77; 18%), Italian (17; 4%), and Romansh (1 or <1%). Five participants did not specify the language spoken.

### Experience in anesthesiology

Experience in years of anesthesiology ranged from 3 years to more than 30 years (median 10 yr, IQR 12–27 yr). The anesthesiologists participating in the survey worked as residents (*n* = 17; 4%), consultants (*n* = 60; 14%), private practitioners (*n* = 51; 12%), senior consultants (*n* = 109; 26%), head doctors (*n* = 102; 24%), and chief of department (*n* = 60; 14%). Nineteen participants (5%) worked in other positions, and four did not specify.

### Self-estimated expertise in PNB

Sixty-three (15%) of the respondents considered themselves experts in PNB, 209 (50%) called themselves experienced users, 119 (28%) had some experience, 25 (6%) had little experience, and four (1%) had no experience at all. Two participants did not provide self-evaluations.

**Figure 1:**
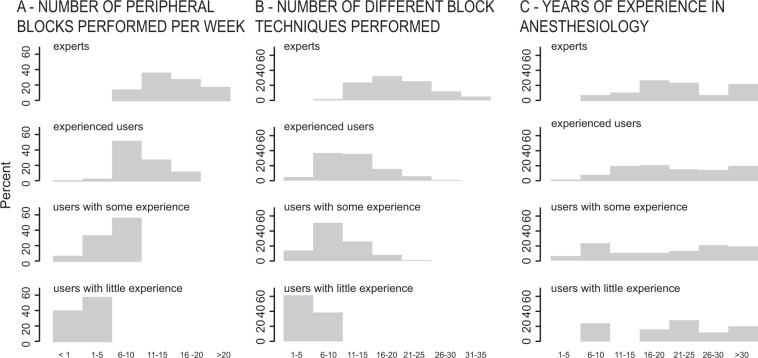
Participants’ self-assessment of their own expertise in the use of peripheral nerve blocks. (**A**) Correlation between expertise and the number of blocks that the participants performed per week. (**B**) Correlation between expertise and the number of different block techniques performed by the anesthesiologists. (**C**) Correlation between expertise and the participants’ experience in anesthesiology in years.

**Figure 2:**
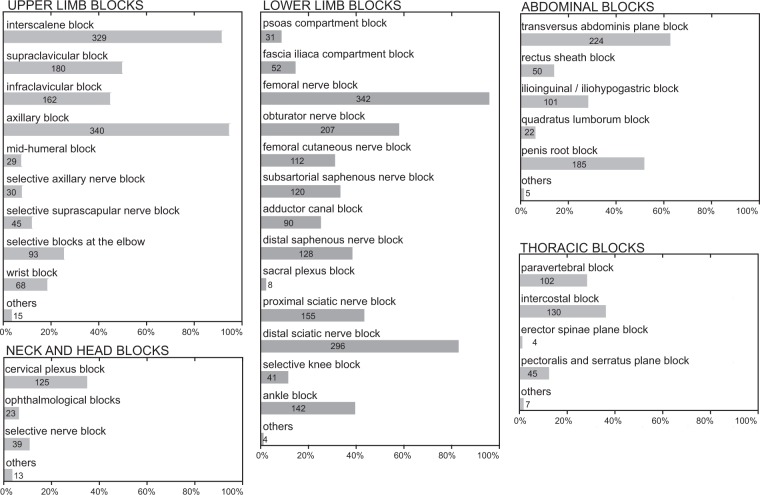
Nerve blocks performed by Swiss anesthesiologists. The figure shows the different types of peripheral nerve block techniques that survey participants were capable of using, broken down by upper limb, lower limb, abdominal, thoracic, and head and neck blocks.

The mean number of different block techniques that anesthesiologists were able to perform was 11.5 ± 5.9 (range 1–32). There was a correlation between self-estimated expertise in PNB and the number of blocks performed per week (ordered logit coefficient 1.14, 95% CI 0.86–1.42, *P* < *0.001*) (Fig. 1A). A correlation could be seen between self-estimated expertise and the number of different block techniques performed by the anesthesiologist (ordered logit coefficient 1.53, 95% CI 0.13 to 0.24, *P* < *0.001*) (see Figs 1B and 2). A less relevant correlation was found between self-estimated expertise and the years of experience in anesthesiology (ordered logit coefficient, 95% CI 0.01 to 0.06, *P* = 0.017) (see Fig. 1C). An ordered logistic regression model was fitted to the data (pseudo r^2^ = 0.29; probability > Chi^2^ < 0.0001).

Femoral nerve blocks, axillary blocks, and interscalene brachial plexus blocks were the three most common PNB’s performed by the participants (Fig. 2). The smallest numbers were reported for erector spinae plane blocks, sacral plexus blocks, and quadratus lumborum blocks. The number of different block techniques performed by the anesthesiologist was derived from information provided by the participants (Fig. 1B). Sixty-five participants did not give information about block techniques.

### Monitoring and sterile barrier precautions

**Figure 3:**
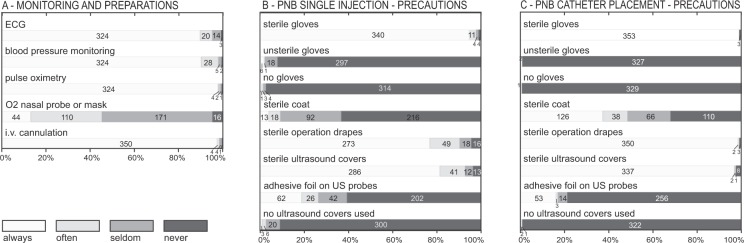
Monitoring and precautions. (**A**) Monitoring and precautions used by anesthesiologists in combination with blocks. (**B**) Sterile barrier precautions for peripheral nerve blocks with single injections. (**C**) Sterile barrier precautions for catheter techniques.

Standard monitoring was regularly used by most anesthesiologists (Fig. 3A). Many did not administer oxygen as a standard, however. Sixty-one of the anesthesiologists did not provide information about the use of monitoring and pre-block preparations. Sterile gloves and sterile ultrasound covers were used for PNB single-shot and catheter techniques by most participants (Fig. 3B,C). For catheter techniques, sterile coats were regularly used by a minority of doctors (Fig. 3B). Sterile coats were less frequently used by anesthesiologists with many years of experience, compared to those with fewer years of experience (ordered logit coefficient −0.05, 95% CI −0.07 to −0.02, *P* < *0.001*; pseudo r^2^ = 0.019; probability > Chi^2^ = 0.02). Sixty-three of the 422 participants did not provide information about their sterile barrier precautions.

### Peripheral nerve localization

**Figure 4:**
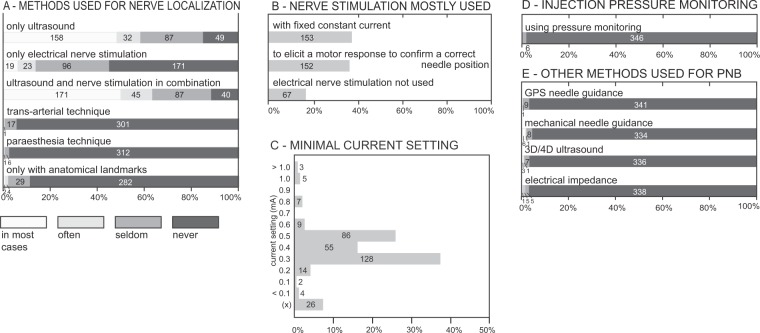
Methods used for peripheral nerve block performance. (**A**) Methods that anesthesiologists reported using for peripheral nerve localization. (**B**) The most frequent use of electrical nerve stimulation. (**C**) Minimal current thresholds (with 0.1 ms impulse duration) normally used by the participants. (x) = participants did not use nerve stimulation. (**D**) Devices for injection pressure monitoring or injection pressure limitation used during peripheral nerve block performance. (**E**) Other methods used by the participants for peripheral block performance.

For the localization of peripheral nerves, a combination of ultrasound and nerve stimulation was used most frequently by the survey participants (Fig. 4A). Only a small proportion of the anesthesiologists did not use ultrasound for PNB at all: 15% of the participants reported that they never used ultrasound as a sole technique for nerve localization, 12% also did not use ultrasound in combination with nerve stimulation. Anesthesiologists with more self-estimated expertise in PNB used nerve stimulation in combination with ultrasound (dual guidance) less frequently than colleagues with less self-estimated expertise (ordered logit coefficient −0.31, 95% CI −0.85 to −0.03, *P* = 0.03; pseudo r^2^ = 0.007; probability > Chi^2^ = 0.05). Anesthesiologists frequently used nerve stimulation with a fixed constant current or to elicit a motor response to confirm a correct needle position (Fig. 4B). Most participants used minimal current thresholds between 0.3 and 0.5 mA (Fig. 4C). Pressure monitoring and guidance systems were very rarely used during PNB performance (Fig. 4D,E). The questions on needle guidance were answered by 358 of the 422 survey respondents.

### Analgesia and anesthesia

**Figure 5:**
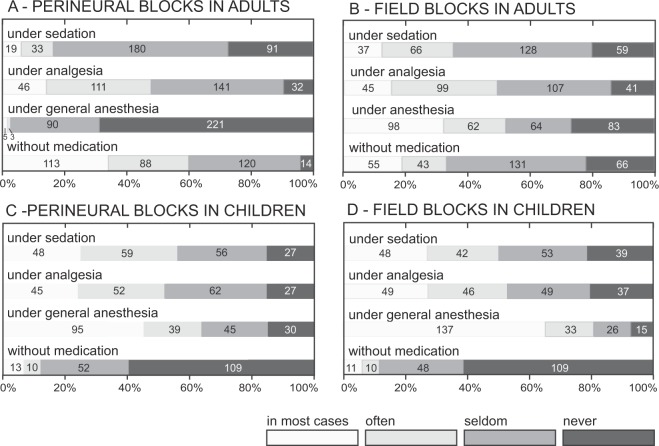
Sedation, analgesia, and anesthesia during peripheral block performance. The figure displays the use of sedation, analgesia and anesthesia in adults during the performance of perineural blocks (with the needle tip in close proximity to a target nerve) (**A**) and during field blocks (**B**). Participants with pediatric experience reported their use of sedation, analgesia and anesthesia in children (0–16 years) during the performance of perineural blocks (**C**) and during field blocks (**D**).

Fourteen (7%) of the anesthesiologists perform PNB in pediatric patients (0–16 years) in most cases, 36 (12%) often, 139 (46%) seldom, and 111 (37%) never. One hundred and twenty-two participants did not provide information about their experience with children. PNB was used with general anesthesia more often in children than in adults (Fig. 5). In both adult and pediatric patients, field blocks were more frequently performed in general anesthesia compared with perineural blocks (with a needle position close to the target nerve). In adult patients, perineural blocks were more frequently performed under general anesthesia by experts in PNB than by colleagues with less self-estimated expertise (ordered logit coefficient 0.69, 95% CI 0.32 to 1.05, *P* < *0.001*; pseudo r^2^ = 0.034; probability > Chi^2^ = 0.0004). In pediatric patients the participants with more years of experience in anesthesiology more frequently performed perineural blocks without general anesthesia, compared with colleagues who had fewer years of anesthesia experience (ordered logit coefficient −0.07, 95% CI −0.10 to −0.04, *P* < *0.001*; pseudo r^2^ = 0.043; probability > Chi^2^ = 0.043). Anesthesiologists in the German speaking part of Switzerland less frequently performed perineural blocks with general anesthesia in pediatric patients (*P* = *0.005*).

The survey participants reported using the following adjuvants: clonidine (*n* = 120, 40%), adrenaline (*n* = 57, 19%), steroids (*n* = 53, 18%), bicarbonate (*n* = 44, 15%), buprenorphine (*n* = 4, 1%), dexmedetomidine (*n* = 4, 1%), fentanyl (*n* = 1, <1%), and nalbuphine (*n* = 1, <1%). One hundred and thirty (43%) anesthesiologists did not use any adjuvant at all. Sixty-six participants did not give information on their use of adjuvants.

### Education and training

When asked about their continuing education and training, the anesthesiologists reported that they participate in courses and seminars (*n* = 250, 84%), hands-on workshops (*n* = 250, 84%), congresses (*n* = 230, 77%), internal clinical training (*n* = 180, 60%), cadaver workshops (*n* = 174, 58%), symposia (*n* = 166, 56%), and other courses (*n* = 9, 3%). Sixty-four participants did not share information about their educational and training opportunities.

### Future trends

Technologies that the participants expected to be routinely used for PNB in ten years were conventional ultrasound (*n* = 299, 99%), 3D or 4D ultrasound (*n* = 136, 45%), electrical nerve stimulation (*n* = 101, 33%), GPS needle guidance (*n* = 84, 28%), and injection pressure monitoring (*n* = 68, 23%). One participant (<1%) believed that none of the techniques mentioned would be routinely used in the future. Sixty-four participants did not answer.

## Discussion

This nationwide study in the central European country Switzerland is the first to report how anesthesiologists prefer to localize peripheral nerves, how they address concomitant safety issues, and the technical approaches they use. To uncover anesthesiologists’ current behavior and technical approaches we asked all members of the Swiss Society for Anaesthesiology and Resuscitation (SSAR/SGAR) to participate in a nationwide survey. The data were analyzed using an ordered logistic regression model.

According to the Helsinki Declaration on Patient Safety in Anaesthesiology, “all institutions providing perioperative anesthesia care […] to patients (in Europe) should comply with the minimum standards of monitoring”^3^. Regional anesthesia textbooks recommend the use of pulse oximetry, ECG monitoring, and intermittent blood pressure measurements^4^. Yet, no guideline specifically states the minimal monitoring to be used in patients with PNB and some of the authors are aware of centers performing regional anesthesia without standard monitoring. Although our data can certainly not be considered as providing evidence, the fact that standard monitoring was regularly used by most anesthesiologists in Switzerland may serve as a “benchmark” for discussions when defining upcoming guidelines^5^.

Hypoxia further lowers the seizure threshold in patients with local anesthetic intoxication. The use of oxygen during PNB performance has also been recommended^4^. In contrast to the use of standard monitoring, less than 50% of Swiss anesthesiologists routinely used oxygen.

The use of electrical nerve stimulation in combination with ultrasound guidance has been recommended to reduce the risk of intraneural needle placement^6–8^. Less than half of the anesthesiologists participating in the survey used ultrasound as a sole technique in most cases. While some authors recommended a minimal current threshold of 0.5 mA for electrical nerve stimulation, others considered using thresholds as high as 0.8–1.0 mA to avoid traumatic needle-to-nerve contact^9–11^. The minimal current thresholds used by the survey participants were often lower than recommended. The German guidelines regarding nerve localization for peripheral nerve blocks state that low threshold currents (<0.5 mA) may potentially increase unintentional intraneural needle placement^12^.

Swiss anesthesiologists frequently use the combination of ultrasound and nerve stimulation to localize peripheral nerves. This differs from other cultural settings such as China, where a 2016 survey revealed that the majority of anesthesiologists rely on anatomical landmarks when performing PNBs^13^. A high share of ultrasound usage amongst younger clinicians helps to avoid maldistribution of local anesthesia, intramuscular needle tip location, and poor choice of both needle angle and insertion site, patterns studied by Sites *et al*.^14^ While the optimal current threshold for nerve stimulation is still under debate, dual guidance is recommended by experts^6,7,9^.

The results regarding the use of sterile gloves and sterile ultrasound covers for PNB single-shot and catheter techniques in our study are in concordance with the findings reported by Fahy *et al*., who found that a majority of anesthesiologists wear sterile gloves (85.7%) and drape the site (57.1%) when performing pediatric caudal blocks^15^. Guidelines published by the German Society of Anaesthesiology and Intensive Care Medicine recommend the use of sterile coats for PNB with catheter placement^8^. In our survey, only 126 of the participants always wore sterile coats for catheter placement. Anesthesiologists with many years of experience used sterile coats less frequently than those with fewer years of experience.

For perineural blocks, general anesthesia was infrequently used in adult patients. This follows many recommendations and guidelines^16^. In pediatric patients, however, general anesthesia is commonly advocated to avoid uncontrolled movements and possibly resulting injuries or complications^17–19^. Many of the participants reported that they did not use general anesthesia in pediatric patients in most cases. More years of anesthesia experience was correlated with a lower likelihood of applying general anesthesia in children undergoing PNBs.

Up to 40% of the participating Swiss anesthesiologists use adjuvants such as clonidine, epinephrine, steroids, bicarbonate, buprenorphine, dexmedetomidine, fentanyl, and nalbuphine for PNBs. However, the evidence for the safe use of adjuvants in peripheral nerve block is limited. In its most recent guidelines, the American Pain Society recommends that clinicians consider the addition of clonidine as an adjuvant for prolongation of analgesia with a single-injection peripheral neural blockade; all other adjuvants are not included in the guidelines^20^.

Currently there are no guidelines with recommendations for developing and maintaining clinical skills. Courses, seminars, and hands-on workshops appear to provide favorable training opportunities, whereas symposia are less frequently considered for training by our cohort. Only a minority of survey participants expect newer techniques such as 3D or 4D ultrasound, electrical nerve stimulation, GPS needle guidance, and injection pressure monitoring to be routinely used in the future.

Our study subjects were all members of SSAR/SGAR, so it remains unknown how our results apply to non-members. Many people working as residents are not members of SSAR/SGAR and are just discovering their work preferences, thus our results may be biased. Because the survey included a considerable proportion of consultants and chairpersons of anesthesia departments, it can be assumed that the results of our study reflect the opinions of key decision makers, clinical leaders and training supervisors in Switzerland, and provide an overview of how regional anesthesia is practiced in this country.

Furthermore, the participation bias might exclude anesthesiologists with little interest (and expertise) in regional anesthesia and explain the high proportion of experienced practitioners.

Although our cohort was limited to members of SSAR/SGAR and the qualitative survey may primarily reflect specific practice in Switzerland descriptively, our data shed light on anesthetists’ current clinical practices and technical approaches to localizing peripheral nerves for regional anesthesia. While our descriptive study does not necessarily reveal new ideas or practices, it presents insights into nationwide professional behavior and, thus, can support clinical judgment in other countries and practice environments^5^. Further, due to its methodological nature, the preliminary, exploratory design of the qualitative survey without formal control might limit the reliability and validity of the survey data in other settings, but the results are an important step towards the definition of recommendations and guidelines for best clinical practice. A future international study using a validated survey will benefit from additionally assessing information about PNB practice distinguishing single injection or catheter techniques.

There is good reason to evaluate self-assessment of the level of expertise with caution. As a validation of the replies of the participants in our survey, we examined the correlation between self-estimated expert level and the number of PNB performed per week, the number of different PNB techniques performed, as well as the years of experience. Remarkably, there is an overlap in the results of these categories. Hence, self-estimation between the participants might vary considerably. An estimation of the “true” expertise would require systematic theoretical and practical tests of the participants. This would be far beyond the scope of our study however.

Switzerland is known for its cultural differences within its language regions. Medical practice is highly influenced by the neighboring countries, Germany, France and Italy. Hence, the results from our survey might reflect regional anesthesia practice from a wider geographical scope in Europe. Yet, analysis with a logistic regression model showed significant differences between respondents with different amounts of anesthesia experience in years (use of sterile coats, blocks in pediatric patients without general anesthesia) and with different self-estimated expertise in PNB (use of dual guidance, blocks under general anesthesia in adults). The regions where the anesthesiologists were located and the main language spoken at their hospitals had only minor effects on these results. In the German speaking part of Switzerland perineural blocks were less frequently performed with general anesthesia in pediatric patients.

In conclusion, our nationwide survey shows anesthesiologists’ preferences for localization of peripheral nerves when performing PNB’s, the technical approaches they use to accurately localize peripheral nerves, and how their practice conforms to current guidelines and recommendations. The use of sterile gloves, sterile ultrasound covers and sterile site drapes appears to be standard practice in our cohort. The high share of respondents who reported employing standard monitoring meets the recommendations of the Helsinki Declaration on Patient Safety in Anaesthesiology. Dual guidance – i.e., the combination of ultrasound and nerve stimulation – appears to be the preferred approach for localizing nerves and providing PNB in Switzerland.

## Methods

Three internationally recognized experts and members of the European Society of Regional Anaesthesia and Pain Therapy (ESRA) from different countries, i.d. Thorsten Steinfeldt (Germany), Urs Eichenberger (Switzerland) and Axel R. Sauter (Norway), developed a qualitative online survey to assess clinical approaches currently used by anesthesiologists in Switzerland as a preliminary, exploratory study as previously described by Vetter *et al*.^21^. Given the nature of a preliminary, exploratory qualitative survey, no formal psychometric reliability or validity tests were performed^21^. For every response simple numerical counts were determined as described before^21^. The online survey with questions about nerve localization tools, safety, and technical approaches used for PNB was prepared in German, French, and Italian versions using the online platform SurveyMonkey (SurveyMonkey Inc., San Mateo, California, USA, www.surveymonkey.com). To ensure validity of the translated questionnaires, these were translated back by other native speakers not invited in the initial translation process. For an English translation of the questionnaire used see Supplemental Digital Content 1.

The 1120 members of the Swiss Society for Anaesthesiology and Resuscitation (SSAR/SGAR) were invited to participate in the survey via an e-mail with links to the three language versions. After two weeks a reminder e-mail was sent. The survey was closed after a four-week period. The survey was anonymous and no identifying information was collected.

The survey contained both single-answer and multiple-choice questions. Rating scales with four possible answers were used in some of the questions, and included either the four options “always”, “often”, “seldom”, and “never” or the four options “in most cases”, “often”, “seldom”, and “never”. Whether the range of answers included “always” or “in most cases” depended on the context of the question.

The survey’s sixth question asked the participants about their experience with PNB. Four participants who answered “no experience” were excluded from the following questions in the survey, and the missing answers were counted as missing data in the results.

### Statistics and data analysis

Five of the questions were selected to undergo statistical analysis. The goal was to determine the effect of self-estimated expertise in PNB, experience in years of anesthesia, and region (main language spoken at the hospital) on the results. Due to the small number of participants from hospitals where Italian and Romansh (Switzerland’s fourth national language) were spoken, we focused on participants who spoke German or French. Responses were analyzed for the following topics: combined use of ultrasound and electrical nerve stimulation (dual guidance), performance of perineural blocks under general anesthesia in both adults and pediatric patients, use of sterile coats for PNB catheter placement, and administration of oxygen to the patient during PNB. Further, the correlation between participants’ self-estimation of their expertise in PNB (“expert”, “experienced operator”, “average experience”, “little experience”) was compared with the number of perineural blocks performed per week, the total number of different blocks performed by the anesthesiologist, and the years of experience in anesthesia. The number of different blocks the anesthesiologists were able to perform was derived from their responses to a list of PNB techniques (24 defined techniques plus “others”). An ordered logistic regression model was fitted to analyze the data. Only statistically significant results are presented. These are shown with ordered logit coefficients, P values, pseudo r^2^ = 0.019; and probability > Chi^2^. A Wilcoxon rank sum test was used to compare language regions (German or French). Statistical results with p values p < 0.05 are displayed as text. More detailed results from all analyses are included as supplemental digital content. Because the study was considered both descriptive and exploratory, no correction was performed for multiplicity.

### Ethics

The project was evaluated and assessed as not requiring authorization by the ethical committee of the canton of Bern (Req-2018-00043).

## Supplementary information

Supplementary Dataset 1

## Data Availability

All data and calculations are available to readers upon request to the corresponding author.
