# Supplementary material for: A Swiss nationwide survey shows that dual guidance is the preferred approach for peripheral nerve blocks
Source: Sci Rep. 2019 Jun 24;9:9178. doi: 10.1038/s41598-019-45700-3 (PMC6591381; doi:10.1038/s41598-019-45700-3)
Supplement: Supplementary file 1 — Supplementary Dataset 1 [file 41598_2019_45700_MOESM1_ESM.pdf]

A Swiss nationwide survey shows that dual guidance is the preferred approach for peripheral nerve blocks

Markus M. Luedi, Vanessa Upadek, Andreas Vogt, Thorsten Steinfeldt, Urs Eichenberger, Axel R. Sauter
Nature Scientific Reports 2019

Supplemental digital content:

- Survey questions, question types, and answers (English translation)
- Detailed results from statistical analysis using ordered logistic regression model and rank sum tests

A Swiss nationwide survey shows that dual guidance is the preferred approach for peripheral nerve blocks

Survey questions, question types, and answers:

Question (1): I am working in a ...
Answers: university hospital / cantonal hospital / regional hospital / district hospital / private hospital / none of the above-mentioned hospitals
Type: single answer multiple choice question

Question (2): In our clinic we speak mostly (language) …
Answers: German / French / Italian / Romansh
Type: single answer multiple choice question

Question (3): I am (male/ female)
Answers: male / female
Type: single answer multiple choice question

Question (4): At the moment I work as a…
Answers: resident / consultant / private practitioner / senior consultant / head doctor / chief of department / other function
Type: single answer multiple choice question

Question (5): My current experience in anaesthesia is ....
less than 1 year / from 1 to 30 years, with consecutive years listed / more than 30 years
Type: dropdown list

Question (6): I would evaluate my expertise in the management of peripheral nerve blocks as ...
Answers: expert / experienced user / average experience / little experience / no experience
Type: single answer multiple choice question

Question (7): How many peripheral nerve blocks do you perform on average per week?
Answers: none / fewer than 1 / 1-5 / 6-10 / 11-15 / 16-20 / more than 20
Type: single answer multiple choice question

Question (8.1 – 8.5): When performing peripheral nerve blocks I take the following precautions:
(8.1) ECG
(8.2) blood pressure
(8.3) pulse oximetry
(8.4) O2 nasal probe or mask
(8.5) intravenous access
Answers: always / often / seldom / never
Type: matrix/rating scale question

Questions (9.1 – 9.4): During the performance of peripheral nerve blocks with perineural needle position (with the needle point in the immediate vicinity of the nerves) my patients are
(9.1) under sedation
(9.2) under analgesia
(9.3) under general anaesthesia
(9.4) without medication
Answers: in most cases / often / seldom / never
Type: matrix/rating scale question

Question (12.1 – 12.4): During the performance of nerve blocks in children with perineural needle position (with the needle point in the immediate vicinity of the nerves) my patients are
(12.1) under sedation
(12.2) under analgesia
(12.3) under general anaesthesia
(12.4) without medication
Answers: in most cases / often / seldom / never
Type: matrix/ rating scale question

Question (13.1 – 13-4): During the performance of nerve blocks in children without perineural needle position (with the needle tip not in the immediate vicinity of the nerves, e.g., field block, TAP block) my patients are
(13.1) under sedation
(13.2) under analgesia
(13.3) under general anaesthesia
(13.4) without medication
Answers: in most cases / often / seldom / never
Type: matrix/ rating scale question

Question (14.1 – 14.5): To localize the nerves during the performance of peripheral nerve blocks with perineural needle position I use …
(14.1) only ultrasound
(14.2) only electrical nerve stimulation
(14.3) ultrasound and electrical nerve stimulation in combination
(14.4) trans-arterial techniques
(14.5) paraesthesia techniques without other aiding devices
(14.6) only with anatomical landmarks
Answers: in most cases / often / seldom / never
Type: matrix/rating scale question

Question (15): I usually use ultrasound in combination with electrical nerve stimulation as follows:
Answers: with fixed constant current / to elicit a motor response to confirm the needle position / I don´t use electrical nerve stimulation
Type: single answer multiple choice question

Question (16): As a minimum current (with a pulse length of 0.1 ms) for a motor current response ("current threshold") before injecting the local anaesthetic I accept …
Answers: consecutive mA listed from 0.1 to 1mA (in steps of 0.1 mA)/ > 1mA/ I usually do not use nerve stimulation
Type: dropdown list

Question (17): I use a device to measure or limit injection pressure (injection pressure monitor) as an additional tool in blocks with perineural needle position
Answers: in most cases / often / seldom / never
Type: single answer multiple choice question

Question (18.1 – 18.4): As an additional tool for the implementation of peripheral nerve blocks I use…
(18.1) GPS needle tracking systems (e.g., electromagnetic or stereotactic)
(18.2) mechanical needle guidance (with a guidance channel attached to the ultrasound probe)
(18.3) 3D/4D ultrasound
(18.4) electrical impedance measurement
Answers: in most cases / often / seldom / never
Type: matrix/rating scale question

Question (19): As adjuvants I use ...
Answers: I do not use adjuvants / bicarbonate / clonidine / dexmedetomidine / steroids (e.g., dexamethasone) / buprenorphine / adrenaline / other substances not on the list
Type: multiple answer question

Question (20.1 – 20.5): During the performance of peripheral nerve blocks with a "single shot" injection technique I use
(20.1) sterile gloves
(20.2) unsterile gloves
(20.3) a sterile coat
(20.4) sterile operation drapes
(20.5) sterile ultrasound covers
(20.6) adhesive foils on ultrasound probes / no ultrasound covers
Answers: always / often / seldom / never
Type: matrix/rating scale question

Question (21.1 – 21.5): During the performance of peripheral nerve blocks with catheter techniques I use
(21.1) sterile gloves
(21.2) unsterile gloves
(21.3) a sterile coat
(21.4) sterile operation drapes
(21.5) sterile ultrasound covers
(21.6) adhesive foils on ultrasound probes / no ultrasound covers
Answers: always / often / seldom / never
Type: matrix/rating scale question

Question (22): I perform the following peripheral nerve blocks in the upper extremities
Answers: no blocks in the upper extremities / interscalene brachial plexus block / supraclavicular brachial plexus block / infraclavicular brachial plexus block / axillary brachial plexus block / mid-humeral brachial plexus block / selective blocks of the axillary nerve / selective blocks of the suprascapular nerve / selective nerve blocks on the elbow / selective nerve blocks on the wrist / other blocks not on the list
Type: multiple answer question

Question (23): I perform the following peripheral nerve blocks in the lower extremities
Type: multiple answer question
Answers: no blockades in the lower extremities / psoas compartment block / fascia iliaca compartment block / femoral nerve block / obturator nerve block / lateral cutaneous femoral nerve block / saphenous nerve block, sub-sartorial / adductors channel block / saphenous nerve block, distal/ sacral plexus block, para-sacral / sciatic nerve block, proximal (trans-, infra-, sub-gluteal) / sciatic nerve block, distal (popliteal) / selective nerve blocks in the area of the knee / selective nerve blocks in the area of the foot / other blocks not on the list.

Question (24): I perform the following peripheral nerve blocks in the area of the head and neck
Answers: no blocks in the head and face area / cervicalis plexus blocks / ophthalmological blocks / selective blocks in the area of the head and face / other blocks not on the list
Type: multiple answer question

Question (25): I perform the following peripheral nerve blocks in the area of the abdomen
Answers: no blocks in the abdominal area / TAP (Transversus Abdominis Plane) block / rectus sheath block / ilioinguinal nerve and iliohypogastric block / quadratus lumborum (QL) block / penis root block / other blocks not on the list
Type: multiple answer question

Question (26): I perform the following peripheral nerve blocks in the area of the thorax
Answers: no blocks in the area of the thorax / paravertebral block / intercostal block / erector spinae plane block (ESP) / pectoralis and serratus plane block / other blocks not on the list
Type: multiple answer question

Question (27): I also use ultrasound for ...
Answer: peripheral vascular punctures / central vascular punctures / the exclusion of a pneumothorax / lumbar and thoracic epidural anaesthesia / interventional pain therapy / identification of the airway / transthoracic echocardiography / transoesophageal echocardiography / FAST (Focused Assessment with Sonography for Trauma) / determination of stomach contents / Doppler method / other areas of application not on the list
Type: multiple answer question

Question (28): On the topic of peripheral nerve blocks, I have taken advantage of the following training and further education opportunities:
Answers: courses and seminars / hands-on workshops / cadaver workshops / conferences / symposia / internal clinic training / other courses
Type: multiple answer question

Question (39): In my opinion, the following methods will be routinely used for peripheral nerve blocks in 10 years ...
Answers: ultrasound / electrical nerve stimulation / injection pressure measurement / needle guidance with GPS tracking / 3D or 4D ultrasound / none of the options mentioned / others
Type: Multiple answer question

Detailed results from statistical analysis using ordered logistic regression model and rank sum tests

Self-estimated expertise

- Correlation with number of blocks performed per week
  (frequencies displayed in Fig. 1a)
  ordered logit coefficient = 1.14, 95% CI 0.86–1.42, P < 0.001)
- Correlation between self-estimated expertise and the number of different block techniques performed by the anesthesiologist
  (frequencies displayed in Fig. 1b and Fig. 2)
  ordered logit coefficient = 1.53, 95% CI 0.13 to 0.24, P < 0.001)
- Correlation between self-estimated expertise and the years of experience in anesthesiology
  (frequencies displayed in Fig. 1c)
  ordered logit coefficient, 95% CI 0.01 to 0.06, *P*=0.017

pseudo r^2 = 0.29; probability>Chi^2 < 0.0001.

Combined use of ultrasound and electrical nerve stimulation (dual guidance)
(frequencies displayed in Fig. 4a)

- Correlation with self-estimated expertise:
  ordered logit coefficient = -0.22, 95% CI -0.51 to 0.05; P = 0.11
- Correlation with years of experience in anesthesia:
  ordered logit coefficient = -0.01, 95% CI -0.04 to 0.01; P = 0.32

pseudo r^2 = 0.005; probability >Chi^2 = 0.15

- Effect of language region: P = 0.36

Use of sterile coats for PNB catheter placement
(frequencies displayed in Fig. 3c)
in relationship to self-estimated expertise:

- Correlation with self-estimated expertise:
  ordered logit coefficient = -0.05, 95% CI -0.31 to 0.22; P = 0.74
- Correlation with years of experience in anesthesia:
  ordered logit coefficient = -0.05, 95% CI -0.07 to - 0.02; P < 0.001

pseudo r^2 = 0.02; probability >Chi^2 = 0.0004

- Effect of language region: P = 0.49

Performance of perineural blocks under general anesthesia in adults
(frequencies displayed in Fig. 5a)

- Correlation with self-estimated expertise:
  ordered logit coefficient = 0.67, 95% CI 0.33 to 1.05; P < 0.001
- Correlation with years of experience in anesthesia:
  ordered logit coefficient = -0.01, 95% CI -0.04 to 0.01; P = 0.43

pseudo r^2 = 0.04; probability >Chi^2 = 0.0004

- Effect of language region: P = 0.56

Performance of perineural blocks under general anesthesia in pediatric patients
(frequencies displayed in Fig. 5c)

- Relationship to self-estimated expertise:
  ordered logit coefficient = -0.19, 95% CI -0.58 to 0.19; P < 0.33
- Relationship to years of experience in anesthesia:
  ordered logit coefficient = -0.07, 95% CI -0.10 to -0.04; P < 0.001

pseudo r^2 = 0.04; probability >Chi^2 < 0.0001

- Effect of language region: P = 0.005
